# Association Between Chronic Pain and Jumping-to-Conclusions Behaviour

**DOI:** 10.1155/prm/6870232

**Published:** 2025-11-04

**Authors:** Nico Schiesewitz, Andreas Schwarzer, Sven Jung, Johannes Forsting, Elena Enax-Krumova

**Affiliations:** ^1^Department of Neurology, BG University Hospital Bergmannsheil gGmbH, Ruhr-University Bochum, Bochum, Germany; ^2^Department of Pain Medicine, BG University Hospital Bergmannsheil gGmbH, Ruhr-University Bochum, Bochum, Germany; ^3^Department of Rehabilitation, BG University Hospital Bergmannsheil gGmbH, Ruhr-University Bochum, Bochum, Germany

**Keywords:** changes in neuropsychological behaviour, chronic limb pain, clinical decision-making, complex regional pain syndrome, jumping to conclusions, neuralgia, peripheral nerve injury

## Abstract

**Objectives:**

A tendency to jumping to conclusions (JTCs) was described in schizophrenia, in functional movement disorders and recently in a mixed group of chronic pain patients and has been discussed to indicate prefrontal dysfunction. This study investigated the tendency to premature decisions (JTC) in patients with complex regional pain syndrome (CRPS), a severe disorder affecting predominantly the distal limb, compared to healthy individuals and those with chronic limb pain from other causes, such as nerve injury or musculoskeletal abnormalities (non-CRPS).

**Methods:**

In the classic ‘beads task', visual stimuli were used to assess evidence-based decision-making ability, followed by a variation with somatosensory stimuli. Thirty patients with CRPS were compared to 23 non-CRPS patients and 30 healthy individuals. Results were related to clinical data such as pain intensity, disease duration, CRPS phenotype based on predominant symptoms (central, peripheral and mixed) and standardized questionnaires evaluating depressive and anxiety symptoms. The main outcome was the number of draws to decision (DTD), i.e., how many beads participants requested before making a final judgement. Group differences were analysed using AN(C)OVA or Kruskal–Wallis tests with Bonferroni-corrected post hoc comparisons, unpaired *t*-tests and chi-squared tests as appropriate. Correlations between beads task performance and clinical parameters were examined using Pearson's or Spearman's analyses, while ANCOVA was applied to control for age, anxiety and depressive symptoms as covariates.

**Results:**

Both CRPS and non-CRPS patients showed a significantly stronger JTC tendency, deciding at a lower DTD (2.63 ± 1.19 and 2.65 ± 1.27, respectively) than healthy controls (5.13 ± 1.92, both *p* < 0.001), without differences between patient groups, and the effect was independent of the CRPS phenotypes. DTD correlated with depressive or anxiety symptoms and age but not with pain intensity or disease duration.

**Conclusion:**

Dysfunctional processes leading to JTC seem to play a role in chronic pain patients and interact with depressive and anxiety symptoms. These interactions may impact treatment outcomes and warrant further investigation.

**Summary**

• Patients with both CRPS and other chronic pain diseases showed a stronger tendency to jumping to conclusions (JTCs) compared to healthy controls.
• Thus, chronic pain seems to lead to a dysfunction of evidence-based decision-making processes, regardless of the origin, duration and intensity of pain and need to be considered for the clinical routine.

## 1. Introduction

The capacity and ability to make decisions influence human life on multiple levels—ranging from small, everyday matters to profound, fundamental events that extend beyond the individual. Various domains of life are affected, including medical and health-related issues. The field of ‘judgement and decision-making' (JDM) has been systematically investigated for several decades [1]. A central framework within this context is the so-called dual-process theory, which differentiates decision-making into two systems: a fast, intuitive *System 1* and a slower, more analytical *System 2* [1]. Although *System 1* is considered more error-prone, both systems are susceptible to mistakes and misjudgements [1]. Within JDM research, particular attention in recent years has been directed towards the tendency to jump to premature conclusions, commonly referred to as ‘jumping to conclusions' (JTCs), especially in relation to disorders encountered in clinical settings.

A premature decision can be interpreted as an alteration in executive functioning, particularly probabilistic reasoning and working memory processes, and has been correlated with dysfunction of the dorsolateral prefrontal cortex (dlPFC) [2]. A tendency to JTCs in the so-called beads task has been described in patients with schizophrenia [3]. This task has been reported to assess impairments in the expectation of sensory impressions [4]. In this process, perception arises bidirectionally from a combination of an expectation based on previous sensory impressions (top-down) and an actual sensory component (bottom-up) [5, 6].

Recently, a tendency to make premature decisions despite a lack of information was reported in a mixed cohort with chronic pain, suggesting that a reasoning bias may play a role in chronic pain through the maintenance of unfavourable neuronal mechanisms [7]. Furthermore, JTC behaviour was also shown in patients with functional movement disorders (FMDs) [8], which can sometimes be a relevant differential diagnosis of complex regional pain syndrome (CRPS) [9].

CRPS is a rare but severe, predominantly unilateral disorder following comparatively trivial trauma or other injuries [10, 11]. It is characterized by generalized motor, sensory, vasomotor and sudomotor dysfunctions, as well as trophic and joint changes predominantly in the distal limb [12]. The aetiology and pathophysiological mechanisms underlying CRPS are not fully understood yet [13, 14]. Symptom severity varies among patients with CRPS, and three distinguished phenotypes have been recently proposed based on the predominant symptoms: ‘central', ‘peripheral' and ‘mixed', categorized by their predominant symptom clusters [15]. Inflammatory signs (temperature asymmetry, asymmetry of skin colour, increased or decreased sweat secretion, oedema and trophic changes) have been reported to indicate a peripheral type, whereas the central type was dominated by symptoms such as allodynia, sensory deficits, motor disturbances or minor injury eliciting CRPS [15].

The central nervous system changes in the context of CRPS include not only maladaptive neuronal activity but also a variety of neuropsychological symptoms [16–18]. These include disturbances of body schema and body image, neglect-like symptoms, impaired left–right recognition and disturbance of spatial body representation [19–21]. Furthermore, psychological symptoms can also influence the CRPS course, such as anxiety, depressive symptoms, affective lability and reduced self-esteem [21–23].

Our study focused on CRPS as a distinct chronic pain syndrome, including a variety of neuropsychological and psychological symptoms, which might influence decision-making in these patients more pronouncedly. Therefore, our primary hypothesis was that CRPS patients display a more pronounced tendency to JTCs compared not only to healthy subjects but also to patients with unilateral chronic pain due to nerve injury or degenerative changes in the musculoskeletal system. The classic ‘beads task' used visually presented stimuli to rule out confounders due to impaired somatosensory perception. Furthermore, a modified version using somatosensory stimuli was performed, as decisions related to somatosensory processing might be influenced in a much more pronounced way than visual stimuli. As secondary aims, we analysed any relation to clinical and psychological parameters such as CRPS phenotype or depressive and anxiety symptoms. Investigating JTC in CRPS is important, as reasoning biases may contribute to maladaptive symptom interpretation and treatment adherence and could therefore have therapeutic implications for pain treatment and rehabilitation programmes, e.g., targeted cognitive interventions.

## 2. Materials and Methods

### 2.1. Participants

This controlled prospective cross-sectional study was carried out from November 2020 until July 2022 as part of the MD thesis of N.S. The local ethics committee approved the study (Medical Faculty, Ruhr University Bochum, Registration No. 20-7050). Informed consent was obtained from all participants according to the Declaration of Helsinki. Patients were recruited from the Department of Pain Medicine and the Department of Rehabilitation at the BG University Hospital Bergmannsheil Bochum. The diagnosis of CRPS of the upper limb was confirmed based on the established clinical diagnostic criteria (‘Budapest criteria'), including both patients without a nerve lesion (Type I) and with a nerve lesion (Type II) [12]. In addition, CRPS patients were classified into three phenotypes based on clinical signs using the cluster-based classification presented by Dimova et al., aiming to reflect different pathophysiological mechanisms [15]. Inflammatory signs (temperature asymmetry, asymmetry of skin colour, increased or decreased sweat secretion, oedema and trophic changes) indicate a peripheral type. In contrast, the central type is defined by symptoms such as allodynia, hyperalgesia, hyperesthesia, motor dysfunction or minor injury as the initiating event [15]. Based on a point system, the central, peripheral and mixed groups were classified. Five inflammatory signs were coded each by −1; four signs of central reorganization were coded by +1. Attributes that were not available were evaluated as 0. Depending on the value of the sum score of all symptoms (peripheral: < 0, mixed: 0 and central: > 0), the patients were assigned to the corresponding group [15].

Patients with localized unilateral pain of the upper limb due to a neurologically confirmed peripheral nerve lesion or due to degenerative changes in the musculoskeletal system were included in the control groups. The healthy subjects were recruited from advertising and among colleagues and were naive to the study hypothesis. All of them fulfilled the published recommendations for inclusion and exclusion criteria of healthy subjects in pain studies [24]. The healthy control group was not matched to the patient groups with respect to demographic characteristics, but age and depressive and anxiety symptoms were considered as possible confounders and were analysed using ANCOVA.

The following exclusion criteria were considered: age < 18 years, lack of informed consent, insufficient knowledge of German, pregnancy and relevant concomitant neurological or psychiatric disease. Possible reasons for secondary exclusion were withdrawal of consent and newly emerged contraindications to inclusion.

### 2.2. Procedures

The examination of the patients took place within one visit. Questionnaires, medical history, clinical examination and the experimental part were carried out by the same examiner (N.S.). The medical history included basic medical data, previous illnesses, current pain medication and an assessment of the Budapest criteria. The clinical examination included the degree of joint movement, the circumferences of the limbs, muscle reflexes and peripheral nerve status.

### 2.3. Beads Task

**Figure 1:**
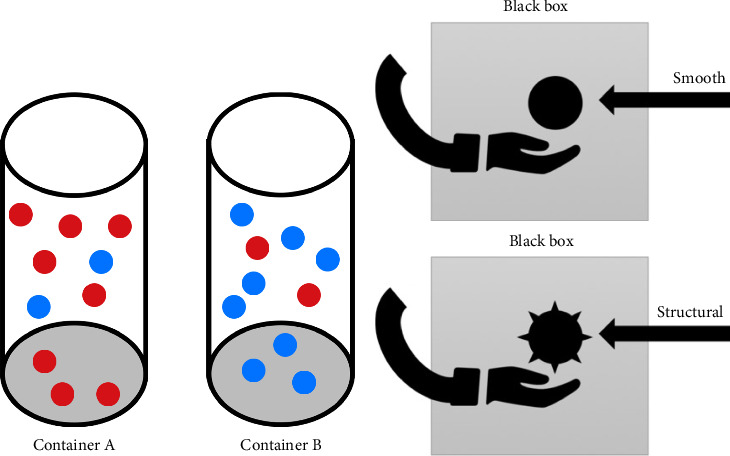
(a) Set up classic beads task: Two equal-looking opaque containers with different amounts of red and blue beads. (b) Set up somatosensory beads task: A black box to expose beads with different surfaces (smooth and structural) to the participants.

The experiment consisted of a visual (classical) beads task and a somatosensory beads task, both employing real physical objects. For the classic beads task, two different opaque containers with red and blue beads were presented to the patients (Figure 1(a)). One of the containers was filled with 80% red and 20% blue beads, whereas the other container included 80% blue and 20% red beads. The task aimed to correctly assign from which container beads were drawn (predominantly red or blue coloured). Therefore, an investigator drew beads sequentially in a certain order and presented them one by one to the participant, who was instructed to decide from which container the beads were being drawn. Participants could request as many beads as they wished before making a final decision. The number of beads drawn before the final decision (draws to decision [DTD]) was measured, and abnormal JTC behaviour was defined as DTD ≤ 2 according to previous literature [3].

To ensure standardization, the order of bead presentation followed fixed, predetermined patterns. The domination of the two colours was predetermined with a standard pattern of red, blue, red, blue, red, red and blue. The participant was shown more red than blue beads in the first trial, with the aim of indicating red as the dominant colour for this trial.

As recommended to enhance reproducibility and reliability, seven runs were conducted in total [25]. Among these, the critical trial (fourth run) was used for primary evaluation, whereas the mean performance across all seven runs was also analysed as recommended. The data-relevant trial with the standard pattern AAABAAAABA was interspersed between three different distraction runs (AABAAABAAA, BAAAABAAAA and AAAABAABAA), as recommended. Therefore, in this pattern, A represented the dominant colour and B represented the less present colour of the beads. Therefore, the first sequence (with red as the dominant colour) was presented as follows: AABAAABAAA (A = red and B = blue). The number of beads the patients wanted to see (DTD) and whether they correctly estimated the dominant colour were recorded.

### 2.4. Somatosensory Variation of the Beads Task

The classical visual beads task primarily tests the influence of perception on visual input stimuli. As patients with CRPS, like those with other chronic pain syndromes, mainly have somatosensory disturbances, a somatosensory modification of the beads task was additionally used (Figure 1(b)).

For this somatosensory modification, two beads with different structures were presented to subjects in a ‘black box' for palpation. This enabled the evaluation based on somatosensory stimuli, excluding visual stimuli. The beads were presented in two different orders according to a standardized scheme. In the first schedule, the smooth bead was presented predominantly (80%), whereas the bead with a structural surface (spiky massage ball) was presented only 20% of the time. In the second schedule, the structural sphere bead was presented predominantly. Like the classic visual beads task, the test participants could ask for any number of beads until they chose one of the programmes. Before the test began, the beads were presented to the patients once visually and tactilely to ensure that the difference between the two beads was apparent to the patients. Analogue to the visual task, there were also seven runs with one standard test pattern and six distraction patterns. Patients were asked to use their affected limb during the testing; the examiner made sure prior to the beginning of the experiment whether patients were able to differentiate between both surfaces and that they were able to tolerate it without pain exacerbation.

### 2.5. Questionnaires

The German version of the Hospital Anxiety and Depression Scale (HADS) [26] Questionnaire was administered to all groups. The subjects in the group with a diseased limb (CRPS and non-CRPS) obtained the German version of PainDETECT Questionnaire [27], Short-Form 12 [28], Disabilities of Arms and Shoulder [29] and Edinburgh Handedness Inventory [30] questionnaires. All questionnaires, including the assessment of pain intensity on the day of examination, were time-locked to be completed immediately before performing the task.

### 2.6. Sample Size Calculation

In a similar study comparing 30 chronic pain patients (aetiology was not reported) with 30 healthy controls, a significant difference was found in a somatosensory variation of the beads task (*p*=0.02 for fewer DTDs; *p*=0.04 for a higher number of premature decision ‘jumpers'; *t*-test or Mann–Whitney *U*-test for ordinal scaled data) [7].

Assuming a power of 90%, an alpha error of 0.05, and the above sample size of *n*1 = *n*2 = 30 (e.g., comparing mean DTD between CRPS patients and the control group), the effect size is *d* = 0.851.

Consequently, a predictive single-factor analysis of variance of the three groups (CRPS, non-CRPS and healthy control) by ANOVA with an effect size of *d* = 0.851, a power of 90% and an alpha error of 0.05 by several cases of *n*1 = *n*2 = *n*3 = 21 could probably demonstrate a significant result and a difference in CRPS patients compared with the other two groups. To investigate possible correlations with pain intensity, disease duration and symptom expression (indicative of a peripheral or central phenotype) within the CRPS group, it was aimed to recruit 30 patients with CRPS and compare them to 30 patients with limb pain of other origin and to 30 healthy control subjects.

### 2.7. Statistical Analysis

Normal distribution was tested by the Kolmogorov–Smirnov test. In the case of normal distribution, the three groups were compared by ANOVA (single-factor group), and in the case of significant differences, post hoc tests for paired comparison were performed using the Bonferroni test. The Kruskal–Wallis test was applied for nonparametric data to test for differences between the three groups, also with Bonferroni as a post hoc test. A significance level of *α* = 0.05 was set. Correlation analysis between the parameters of the beads task and clinical data was performed using Pearson's correlation analysis in the case of normal distribution or Spearman's correlation analysis in the case of non-normal distribution or ordinal data. Correlation levels were tested based on the available data of all three study groups; only correlations with the CRPS phenotype were calculated for the CRPS group. Chi-squared tests were used to assess gender (male vs. female) and JTC behaviour, which was defined as dichotomic (JTC behaviour DTD < 2 and non-JTC behaviour DTD > 2). A post hoc Bonferroni analysis was performed using a corrected alpha of *α* = 0.008333. ANCOVA with age, anxiety and depressive symptoms as covariates was used to examine group differences considering the abovementioned confounders.

## 3. Results

### 3.1. Clinical Data

According to the performed sample size calculation, 30 patients with CRPS and 23 patients with unilateral pain of other aetiology (due to a painful peripheral nerve lesion or degenerative changes in the musculoskeletal system) were compared to 30 healthy control subjects. Clinical and demographic data are shown in Table 1. The non-CRPS patient group comprised 23 instead of 30 participants, as recruitment was limited by the inclusion criteria and the time frame of the study. Therefore, the final sample size of 23 patients was used for the analyses.

Regarding age, there was a significant difference between the healthy controls and the CRPS group (Kruskal–Wallis test and post hoc Bonferroni: CRPS vs. healthy controls *p* < 0.001 and non-CRPS vs. healthy controls *p*=0.084).

All CRPS patients showed symptoms according to the Budapest criteria, with 90% having sensory abnormalities. When dividing the CRPS group based on their clinical phenotype [15], 18 patients showed a peripheral phenotype with more negative sensory symptoms. There were seven patients with a central phenotype and more positive sensory symptoms (Table 2). The remaining five patients showed positive and negative symptoms with a balanced score and were classified as mixed phenotype [15].

Comparing both patient groups, there was no significant difference in disease duration. The average and maximal pain intensity of the CRPS was significantly lower than in the non-CRPS group (Table 1). Both patient groups showed significantly higher HADS scores than healthy controls (Kruskal–Wallis test and post hoc Bonferroni for HADS-A: CRPS vs. healthy controls *p*=0.004 and non-CRPS vs. healthy controls *p* < 0.001; Table 1). Both patient groups received pain medications, which are described in detail in Table 1.

### 3.2. Classic Visual Beads Task

**Figure 2:**
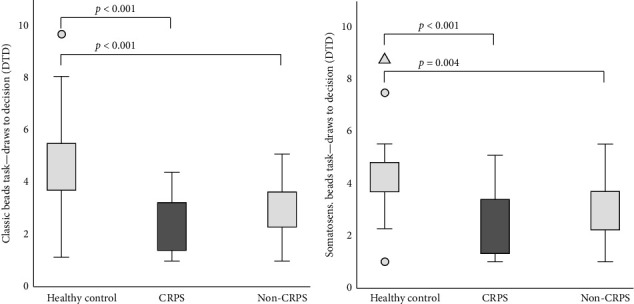
(a) Classic beads task. Mean draws to decision (DTD) in multiple trials (seven runs) from patients with CRPS, chronic limb pain of other aetiology (non-CRPS) and healthy subjects (significant differences between patient groups and healthy controls). (b) Somatosensory beads task. Mean draws to decision (DTD) in multiple trials (seven runs) from patients with CRPS, chronic limb pain of other aetiology (non-CRPS) and healthy subjects (significant differences between patient groups and healthy controls). CRPS, complex regional pain syndrome; DTD, draws to decision.

For all participants, values for the classical task were available. Both patient groups showed significantly lower DTD in a classic beads task than healthy controls (Kruskal–Wallis test and post hoc Bonferroni—single trial: CRPS vs. healthy controls: *p* < 0.001 and non-CRPS vs. healthy controls: *p* < 0.001; Figure 2(a), Table 3). There was no significant difference in DTD between the CRPS and non-CRPS groups. Applying the mean of the multiple runs (seven trials in total) also showed a significant difference in univariate ANOVA for DTD between both patient groups and healthy controls, but without a difference between the CRPS and non-CRPS groups. When comparing the number of participants with abnormal JTC behaviour (cutoff DTD ≤ 2 in a single trial), a significant difference between the groups could be found (Pearson's chi-square *p*=0.019). In post hoc Bonferroni-corrected analysis, we found that significantly fewer individuals in the healthy control group showed JTC behaviour than statistically expected (post hoc Bonferroni for Pearson's chi-square: healthy controls with JTC behaviour *p*=0.005; z-score −2.80). JTC behaviour was found in 43% of the patients with CRPS and 44% of the non-CRPS patients compared to 13% of the healthy controls (Table 3). CRPS phenotypes (central, mixed and peripheral) did not differ from each other regarding the DTD results (Table 3).

### 3.3. Somatosensory Beads Task

For all participants, values for the somatosensory variation of the beads task were available. All patients tolerated the task using the affected limb.

The DTD was significantly lower in both patient groups compared to the healthy controls (Kruskal–Wallis test and post hoc Bonferroni—single trial: CRPS vs. healthy controls: *p* < 0.001 and non-CRPS vs. healthy controls: *p*=0.004; ANOVA and post hoc Bonferroni—multiple trials: CRPS vs. healthy controls: *p* < 0.001 and non-CRPS vs. healthy controls: *p* < 0.001; Figure 2(b) and Table 3). There were no differences between the CRPS and non-CRPS groups (Table 3). Again, a significant difference between the groups could be found when comparing individuals with JTC behaviour (Pearson's chi-square *p*=0.012). The post hoc Bonferroni correction showed that significantly more individuals in the CRPS group showed JTC behaviour than statistically expected (post hoc Bonferroni for Pearson's chi-square: CRPS with JTC behaviour *p* < 0.001; z-score 2.64). About 53% of CRPS patients and 35% of non-CRPS patients showed JTC behaviour in the somatosensory adjustment, whereas it was the case in only 17% of healthy controls. There was no difference between the CRPS phenotypes (Table 3).

### 3.4. Correlations Between DTD, Clinical Parameters and Questionnaires

The DTD did not correlate with pain intensity or duration of the disease. There were significant correlations between DTD and level of anxiety and depressive symptoms assessed by the HADS score. Likewise, a correlation between DTD and age could be found. The scores of the PainDETECT, Short-Form 12 and Disabilities of Arms and Shoulder questionnaires showed no correlation to the DTD. A more detailed overview is shown in Table 4.

### 3.5. Correctness of Container Prediction

A pairwise comparison of the mean total number of correctly stated containers showed a significant difference between the CRPS group and the healthy controls in the somatosensory task (Kruskal–Wallis test and post hoc Bonferroni—individual survey: CRPS vs. healthy controls: *p* < 0.001). The CRPS group was significantly more likely to report a false prediction of the containers. In contrast, no difference was found between the CRPS group and the non-CRPS group. The classic beads task showed no significant difference between any of the groups.

### 3.6. Influence of Possible Confounding Factors

To exclude the influence of anxiety, depressive symptoms and age as covariates on the analyses conducted, an ANCOVA was performed, including the mean DTD of the classic and somatosensory variants. Nevertheless, there was a significant difference between all three groups for all covariates in both the classic and the somatosensory tasks.

## 4. Discussion

### 4.1. Summary of Results

To summarize, our findings support the hypothesis that chronic pain patients show a tendency to JTCs. Both patient groups showed a significantly lower number of DTD than healthy controls. Significant differences between observed and expected frequencies were found when examining the dichotomous JTC behaviour with a cutoff set to DTD ≤ 2. In the classic beads task, significantly fewer individuals in the healthy control group showed a JTC than expected. In the somatosensory variation, significantly more CRPS patients showed JTC behaviour than expected. However, there was no difference between the CRPS and non-CRPS groups despite the different entities and the significantly different average pain levels. Furthermore, pain intensity and DTD did not correlate. However, DTD correlated with age, anxiety and depressive symptoms, although group differences remained after considering those confounders.

### 4.2. Rationale for the Somatosensory Task

In the previous study of chronic pain patients [7], only the somatosensory task using vibrotactile stimuli detected significant group differences vs. healthy controls. Therefore, a somatosensory adjustment of the beads task was also included in the present study, focussing on surface differences (smooth vs structural bead). Good tactile discrimination of 2D objects by CRPS patients could be previously demonstrated despite sensory dysfunction [31].

Furthermore, all pain patients could use the affected limb for palpation and to detect the structural differences in the beads in the pretesting. Therefore, we do not expect that the results of the somatosensory task would be affected by possibly impaired sensory function. We adapted the somatosensory modification to the classical beads task as closely as possible. The enhancement of robustness was also secured due to the increase in the number of possible trials during the experiment. Furthermore, the comparative evaluation in relation to one trial and the mean of seven should maximize reliability and reproducibility [25].

### 4.3. Underlying Mechanisms of Decision-Making

The results suggest that in chronic pain patients, a disproportionate weighting of sensory and visual influences might be part of the process that leads to early decision-making. The influence of persistent pain on the thalamocortical loop could underline this theory, as CRPS patients showed stronger functional connectivity between the thalamus and the somatosensory cortex in a tactile experiment [20]. Although there was no significant difference between the classic and somatosensory beads tasks, inadequate processing of both sensory and visual stimuli may hint at an influence on the JTC behaviour.

Patients with neurosurgical excisions in the dlPFC showed significantly stronger JTC behaviour [2]. The prefrontal cortex is regarded as a central point in the further processing of heterogeneous functional signals [32–35]. A reduction in grey matter in the dlPFC was found in chronic pain patients [36–39]. The dlPFC serves as a link between pain processing and cortical structures [39]. It inhibits the thalamus and insula and leads to a reduction in pain [40]. The increased JTC behaviour in both patient groups in our study supports the previous assumptions [7] of an existing association of maladaptive processes in the dlPFC with pain processing, which does not seem to be specific to CRPS. Interestingly, previous research on multisensory and sensorimotor integration found no evidence that these were impaired in CRPS [41, 42]. Despite the limitation of the small CPRS subgroups, no significant differences could be detected also between different phenotypes based on predominant symptoms either [15]. In the current cross-sectional analysis, JTC behaviour was not correlated with subjective pain intensity or duration of the disease. However, it remains unclear whether reducing pain intensity through therapeutic measures might reduce the level of cognitive dysfunction and JTC behaviour in the long term.

### 4.4. Cognitive Function and Chronic Pain

Dysfunctional cognitive processes or cognitive decline could partly be observed in connection with chronic pain [43]. However, meta-analyses showed inconclusive results depending on the test procedure [43]. The analysis of the Short-Form 36 Health Survey Questionnaire (SF-36) mental component summary, Montreal cognitive assessment, performance validity testing or operation span revealed a correlation between chronic pain and mental decline. In contrast, tests such as the International Classification of Diseases and Related Health Problems classification, the Mini-Mental State Examination or the Repeatable Battery for the Assessment of Neuropsychological Status memory component represented contradictory results [43]. At this point in time, there are very few data sets that record decision-making in general as a parameter that can be influenced. In particular, with increasing age, persistent pain seems to have a high impact on cognitive performance [43]. Furthermore, individual entities with chronic or persistent pain, such as fibromyalgia, show increased neuronal recruitment to perform cognitively demanding tasks compared to healthy controls [44].

### 4.5. Clinical Relevance

The findings of the above-described early decision-making have implications for clinical practice. JTC behaviour could influence patients' choices regarding medical procedures. For instance, during medical patient education, e.g., regarding treatment options, chronic pain patients could be prone to making premature decisions. In CRPS, a notable proportion of patients consider an amputation of the affected limb as a potential treatment hoping for pain reduction [45]. However, there is insufficient evidence to confirm that such an approach improves symptoms, prevents the recurrence of CRPS or enhances quality of life [46]. Therefore, not only irreversible treatment decisions like these but also other invasive approaches should be carefully and thoroughly evaluated with patients, even in cases of long-term, treatment-resistant CRPS disorders, based on the results of this study.

This may affect the long-term impact of CRPS on limb function and work status [47] and should be central when counselling and treating chronic pain patients in general. New counselling approaches regarding ‘shared-decision-making', such as the ‘three-talk model' by [48], could offer great benefits through structured rational and situational counselling and patient involvement. Also, further approaches to integrating patient experiences into the education and treatment of CRPS patients could also help identify and address maladaptive cognitive processing mechanisms [49].

### 4.6. Limitations

Despite the standardized procedures of the study, several factors may have influenced the results. For example, time pressure during the examination or lack of attention due to motivational factors could have reduced the number of beads requested across groups, leading to an artificial increase in apparent JTC behaviour. Similarly, increased levels of pain on palpation might have biased participants towards premature decisions and lower DTD values. However, the latter is contradicted by the equally significant results of the classical visual-only beads task, which should have been less influenced by the current pain intensity. Also, the pain was not investigated in most of the studies with the application of the beads task.

Educational level or personality traits may also have influenced reasoning strategies, although the extent of these effects on the beads task remains unclear.

To date, no studies are available on the degree to which educational status affects the beads task. However, a previous study on 19 patients after neurosurgical excision of the prefrontal cortex in comparison with patients with diagnosed attention-deficit/hyperactivity disorder (ADHD) and 25 healthy controls showed that an internal higher impulsivity does not influence the beads task as much as predicted [2]. They concluded that a lack of concentration skills does not automatically lead to premature decision-making in the beads task. As the healthy control group was not matched to the patient groups in terms of demographic characteristics, these variables may have influenced the results. In addition, anxiety and depressive symptoms can influence the result as correlating variables, as both showed significant correlations to the DTD. In contrast to this, no correlation between anxiety and depressive symptoms and DTD was found in Parkes' comparative study [7]. Although the patient groups differed from healthy controls in terms of age, anxiety and depression, the ANCOVA results demonstrated that significant between-group differences in DTD remained even after statistically controlling for these covariates. This indicates that the observed effects cannot be fully explained by differences in affective symptoms or age but rather reflect group-specific findings. Nevertheless, as anxiety and depression represent relevant influencing factors associated with JTC and differ between clinical and healthy populations, a further limitation of the present study is the absence of a control group characterized by anxiety or depression without pain. Future research should therefore include such a group to disentangle the effects of pain from those of comorbid affective disorders.

However, the present cross-sectional study design does not allow differentiation of whether pain triggers premature decision-making or whether this tendency to JTCs predisposes the development of chronic pain. Future studies with a longitudinal design could address this. The relationship between the degree of maladaptive plasticity and the dysfunction of specific cortical areas is also unclear.

Furthermore, indeed it is possible that some participants with a JTC tendency happened to make a correct choice by chance when answering the task, which might question its meaningfulness. However, this does not affect the validity of the JTC effect itself, as the phenomenon is defined by the number of draws taken before a decision is reached rather than the accuracy of the final choice. The task is therefore designed to assess reasoning style under uncertainty rather than decision correctness. Accordingly, the main finding remains that patients demonstrated a stronger tendency to JTCs, independent of whether the final decision happened to be correct.

## 5. Conclusion

In summary, both CRPS patients and patients with unilateral limb pain of other origin showed lower numbers of DTD compared to healthy controls. Despite the previously reported central maladaptive changes in CRPS, no significant differences in premature decision-making were observed compared to other chronic pain syndromes, although this should be interpreted with caution given the limited statistical power. In conclusion, dysfunctional processes leading to JTC behaviour seem to play a role in chronic pain patients regardless of the origin, duration and intensity of pain and need to be considered for the clinical routine.

## Figures and Tables

**Table 1:** Clinical data.

	CRPS	Non-CRPS	Healthy control	*p*	Post hoc paired comparison
	(*n* = 30)	(*n* = 23)	(*n* = 30)		
Male, *n*	18 (60%)	13 (43%)	15 (65%)	0.232^a^	
Age, years	36 ± 15	54.23 ± 9	47.6 ± 13	< 0.001^b^	CRPS vs non-CPRS: 0,180^d^ CRPS vs. HC: < 0.001^d^ Non-CRPS vs. HC: 0,084^d^
Handedness (right)	26 (86%)	21 (91%)			
Right limb affected	11 (36%)	13 (56%)			
CRPS type					
Type 1	22 (73.3%)				
Type 2	8 (26.7%)				
Disease duration, weeks	18 ± 23	75 ± 172		0.293^c^	
Pain intensity during the examination start	3 ± 2	4 ± 3		0.075^c^	
Pain intensity during the examination end	3 ± 2	4 ± 3		0.082^c^	
Average pain intensity	5 ± 2	6 ± 2		0.013^c^	
Maximum pain intensity	6 ± 2	8 ± 2		0.031^c^	
PainDETECT score	15.8 ± 6.7	18.6 ± 7.2			
Precipitating event					
Fracture	19 (63.3%)	10 (43.5%)			
Soft tissue trauma	9 (30%)	10 (43.5%)			
Surgery	1 (3.3%)	0 (0%)			
Entrapment syndrome	1 (3.3%)	1 (4.3%)			
Other	0 (0%)	2 (8.7%)			
Current medication, *n* (%)					
NSAID/dipyrone	20 (66.7%)	15 (65.2%)			
Moderate opioids	4 (13.3%)	2 (8.7%)			
Strong opioids	1 (3.3%)	0 (0%)			
Tricyclic antidepressants	4 (13.3%)	5 (21.7%)			
SSRI/SNRI	2 (6.7%)	0 (0%)			
Anticonvulsant	8 (26.7%)	6 (26.1%)			
HADS-A	7.7 ± 4.28	8.7 ± 5.04	4.3 ± 2.59	< 0.001^e^ (*F* = 9,3)	CRPS vs non-CPRS: > 0.99^d^ CRPS vs. HC: 0.004^d^ Non-CRPS vs. HC: < 0.001^d^
HADS-D	7.3 ± 3.87	7.6 ± 4.52	3.4 ± 2.34	< 0.001^b^	CRPS vs non-CPRS: > 0.99^d^ CRPS vs. HC: < 0.001^d^ Non-CRPS vs. HC: < 0.001^d^

*Note:* All data are given as mean, standard deviation or absolute numbers. All pain ratings are given as NRS 0–10. Multiple responses were possible for the current medication. PainDETECT Questionnaire (Freynhagen et al., 2006); Hospital Anxiety and Depression Scale (HADS)-A/D [26].

Abbreviations: NSAID, nonsteroidal anti-inflammatory drug; SNRI, selective noradrenaline reuptake inhibitor; SSRI, selective serotonin reuptake inhibitor.

^a^Chi-squared test.

^b^Kruskal–Wallis test.

^c^Mann–Whitney U-test.

^d^Post hoc Bonferroni.

^e^ANOVA.

**Table 2:** Signs and symptoms.

	CRPS
	*n* = 30
Patients with central symptom categories	
Sensory	27 (90%)
Allodynia	7 (23.3%)
Motor	29 (96.7%)
Minor injury	0 (0%)
Patients with peripheral symptom categories	
Trophic	14 (46.7%)
Sweating	20 (66.7%)
Temperature	17 (56.7%)
Colour	12 (40%)
Oedema	21 (70%)
CRPS clinical phenotypes [15]	
Central	7 (23.3%)
Peripheral	18 (60%)
Mixed	5 (16.7%)

*Note:* Distribution oriented at the Budapest criteria [12] and CRPS phenotypes from [15]. All data are given as absolute numbers with percentages in parentheses.

**Table 3:** Parameters of the classic and somatosensory beads task [4].

**A**				
	**CRPS (*n* = 30)**	**Non-CRPS (*n* = 23)**	**Healthy control (*n* = 30)**	

Classic beads task				
Single trial DTD	2.63 ± 1.19	2.65 ± 1.27	5.13 ± 1.92	< 0.001^a^
Multiple trial DTD	2.56 ± 1.05	2.91 ± 1.17	4.69 ± 1.67	< 0.001^b^ (*F* = 20.96)
JTC behaviour, n	13 (43.3%)	10 (43.5%)	4 (13.3%)	0.019^e^
Somatosensory beads task				
Single trial DTD	2.4 ± 1.22	2.87 ± 1.22	4.57 ± 1.87	< 0.001^a^
Multiple trial DTD	2.51 ± 1.2	2.89 ± 1.22	4.34 ± 1.46	< 0.001^b^ (*F* = 16.03)
JTC behaviour	16 (53.3%)	8 (34.8%)	5 (16.7%)	0.012^e^
JTC behaviour post hoc Bonferroni for Pearson's chi-square				Bonferroni-corrected alpha = 0.008333
Classic beads task				
*z*-Score	1.58^f^	1.31^f^	−2.80^f^	
*p*-Value	0.110	0.194	0.005	
Somatosensory beads task				
*z*-Score	2.64^f^	−0.02^f^	−2.63^f^	
*p*-Value	0.008290	0.985	0.0085	

**B**				
	**CRPS vs. healthy control**	**Non-CRPS vs. healthy control**	**CRPS vs. non-CRPS**	

Classic beads task				
Single trial DTD (1 out of 7)	< 0.001^d^	< 0.001^d^	1.000^d^	
Single trial DTD (3 out of 7)	< 0.001^d^	< 0.001^d^	1.000^d^	
Multiple trial DTD	< 0.001^c^	< 0.001^c^	1.000^c^	
Somatosensory beads task				
Single trial DTD	< 0.001^d^	0.004^d^	0.661^d^	
Multiple trial DTD	< 0.001^c^	< 0.001^c^	0.915^c^	

**C**				
**CRPS phenotypes**	**Central (*n* = 7)**	**Peripheral (*n* = 18)**	**Mixed (*n* = 5)**	

Classic beads task				
Single trial DTD	2.71 ± 1.4	2.5 ± 1.1	3 ± 1.4	0.720^a^
Multiple trial DTD	2.39 ± 1	2.46 ± 1	3.11 ± 1	0.435^b^ (*F* = 0.859)
Somatosensory beads task				
Single trial DTD	2.86 ± 1.6	2.11 ± 1	2.8 ± 1.5	0.426^a^
Multiple trial DTD	2.65 ± 1.3	2.33 ± 1.2	2.97 ± 1	0.556^b^ (*F* = 0.599)

*Note:* (A) Grouping made according to examination groups (CRPS, non-CRPS and healthy control) and Pearson's chi-square with post hoc Bonferroni correction. (B) Significance values in paired comparison of groups (*p*). Effect size *r* given in parentheses. (C) Division of the CRPS group into central, peripheral and mixed according to [15]. All data are given as mean, standard deviation or absolute numbers with percentages in parentheses.

^a^Kruskal–Wallis test.

^b^ANOVA.

^c^ANOVA and post hoc Bonferroni.

^d^Kruskal–Wallis and post hoc Bonferroni.

^e^Pearson's chi-square test.

^f^Crosstabs-adjusted residuals.

**Table 4:** Correlation of multiple trial means of DTD to assessed clinical parameters.

	Classic beads task		Somatosensory beads task	
	Multiple trial DTD		Multiple trial DTD	
	Correlation coefficient	Significance	Correlation coefficient	Significance
CRPS phenotype^a^	−0.143	0.450	−0.226	0.229
Average pain intensity^a^	0.125	0.371	0.121	0.389
Disease duration, weeks^a^	0.110	0.937	0.034	0.809
HADS-A score^b^	−0.270	0.014	−0.244	0.027
HADS-D score^a^	−0.226	0.039	−0.240	0.029
Age, years^a^	−0.485	< 0.001	−0.479	< 0.001
SF-12				
PCS^b^	0.043	0.762	0.127	0.365
MCS^b^	0.048	0.733	0.117	0.403
DASH^b^	−0.078	0.581	−0.261	0.059
PainDETECT^b^	0.063	0.654	−0.049	0.727

*Note:* All data are correlation coefficients (*r*) or significance levels (*p*). All available data from all groups were included. HADS-A/D score, Hospital Anxiety and Depression Scale-A/D score; SF-12, Short-Form 12 Questionnaire; DASH, Disabilities of Arms and Shoulder; PainDETECT, PainDETECT Questionnaire score.

Abbreviations: MCS, mental component scale; PCS, physical component scale.

^a^Spearman's correlation.

^b^Pearson's correlation.

## Data Availability

The data that support the findings of this study are available on request from the corresponding author. The data are not publicly available due to privacy or ethical restrictions.

## Nomenclature

CRPS: Complex regional pain syndrome
DTD: Draws to decision
dlPFC: Dorsolateral prefrontal cortex
FMD: Functional movement disorders
JTC: Jumping to conclusions
